# Formation and Investigation of Mechanical, Thermal, Optical and Wetting Properties of Melt-Spun Multifilament Poly(lactic acid) Yarns with Added Rosins

**DOI:** 10.3390/polym14030379

**Published:** 2022-01-19

**Authors:** Evaldas Bolskis, Erika Adomavičiūtė, Egidijus Griškonis

**Affiliations:** 1 Faculty of Mechanical Engineering and Design, Kaunas University of Technology, Studentu Str. 56, 51424 Kaunas, Lithuania; erika.adomaviciute@ktu.lt; 2 Faculty of Chemical Technology, Kaunas University of Technology, Radvilenu pl. 19, 50254 Kaunas, Lithuania; egidijus.griskonis@ktu.lt

**Keywords:** PLA, melt-spinning, rosin, multifilament yarns

## Abstract

One method for adding enhancing properties to textile materials is the insertion of natural ingredients into the textile products during the manufacturing or finishing process. The aim of this research is to investigate the formation of biodegradable melt-spun multifilament Poly(lactic acid) (PLA) yarns with different contents (i.e., 5%, 10%, and 15%) of natural material–rosin, also known as colophony. In this study, multifilament yarns were successfully formed from PLA and a natural substance–pine rosin by melt-spinning them at two different draw ratios (i.e., 1.75 and 2.75). The results indicated that a 1.75 draw ratio caused the formation of PLA and PLA/rosin yarns that were brittle. The presence of rosin (i.e., 5% and 10%) in multifilament yarns decreased the mechanical properties of the PLA/rosin melt-spun multifilament yarns’ tenacity (cN/tex), breaking tenacity (cN/tex), and tensile strain (%) and elongation at break (%) and increased absorbance in the entire UV region spectra. In addition, the melting point and degree of crystallinity decreased and there was an increase in the wetting angle compared with pure PLA multifilament. The investigation of melt-spun yarns with Raman spectroscopy proved the presence of rosin in PLA melt-spun yarns.

## 1. Introduction

Melt-spinning is one the most economical and widely used yarn spinning processes. This process has many advantages compared to wet or dry-spinning: simplicity, a high productivity production line, and low production cost. The two most important reasons for its use are that (i) there is no need to use solvents in the production of polymer yarns due the fact that melt-spun yarns are ideal for medical application; (ii) it is possible to use additives and form multicomponent yarns with various functionalities [1,2]. The important process variables that influence the structure and properties of the melt-spun filaments are extrusion temperatures; mass throughput per spinneret hole; cooling conditions; size and shape of the spinneret holes; spin line length, and take-up velocity of the filaments or filament draw ratio [3].

Many polymers can be used in melt-spinning processes such as poly(ethylene terephthalate), polyurethanes, polyolefins, and polyamides [4]. However, using some of them (i.e., petroleum-derived polymers) in this process and their waste are a major problem in terms of global pollution.

Poly(lactic acid) (PLA) is an aliphatic polyester synthesized from natural resources (most often from corn and sugar cane) with a production capacity of approximately 400,000 tons in 2020 [5,6]. This promising thermoplastic biopolymer possesses various properties such as biodegradability that can be conducted in aqueous or biological mediums, typically at 37 °C and at approximately pH 7 in vitro and in vivo. Moreover PLA is biocompatible and bioabsorbable, has good mechanical properties, and is widely used for medical applications [7,8,9,10]. The properties of PLA can be modified by blending, plasticization and/or reactive processing. Blending a polymer with other nanoparticles or polyolefin polymers is a simple method to potentially improve the properties of pure polymers [11]. Ferreira et al. [12] examined manufactured Poly(d,l-lactic acid) (PDLLA) monofilament yarns containing N,N-diethyl-3-methylbenzamide (DEET). DEET is a popular and widely available mosquito repellent, and it is a method of protection against malaria. It was estimated that with 10% and 20% of DEET, it is possible to form monofilament PDLLA yarns. The ultimate strength of PDLLA multifilament yarns decreased by approximately twice when used with 10% DEET and ten times with 20% DEET. Barral and co-authors [13] investigated the effect of a melt-spinning process-formed mono- and multifilament. It was produced from a polymer blend of PLA and polycaprolactone (PCL). Both polymers are immiscible, so it was investigated how this influences the filament structure and degradation behavior A large range of these blends from 90/10 to 60/40 wt% PLA/PCL was processed via single filament extrusion (diameter monofilament: ∅ ≈ 1 mm) and multifilament melt-spinning (80 filaments: 50–70 µm each). It was estimated that the highest elongation at break of the extruded PLA/PCL monofilament was when 30% of the PCL was blended. Multifilament yarns’ values of elongation at break decrease no matter what amount of PCL is added to the polymer mixture. In addition, it was estimated that the fiber morphology had an impact on the accessibility of an immiscible blend. Comparing PLA_90_/PCL_10_ monofilament and multifilament yarns, it was estimated that a part of the PCL in the monofilament yarn was trapped in the PLA matrix and not all PCL were accessibly solvent. Meanwhile, in multifilament PLA/PCL, the blend containing at least 10 wt% of PCL, a very high PCL accessibility was seen. This was influenced by fiber processing parameters and an increased specific surface and surface-to-volume ratio of multifilaments. Alliota et al. [14] investigated the influence of different compositions of PLA/poly(butylene succinate-co-adipate) (PBSA) (80/20 and 60/40 wt%) on multifilament yarn properties. It was estimated that different blends showed different morphologies. It was observed that an 80/20 wt% blend possessed a morphology where the PBSA particles were dispersed within the PLA matrix, but in the 60/40 wt% blend, a co-continuous structure was found. Pivsa-Art et al. [15] analyzed bicomponent multifilament yarns that consisted of a neat, recycled polyethylene terephthalate (RPET) core and RPET/nano-TiO_2_ shell. The nano-TiO_2_ content in the shell varied between 1 and 3 wt%. They found that the core/shell ratio had no effect on the mechanical properties, namely, tenacity and elongation at break. Meanwhile, the tenacity and elongation at break were positively correlated with TiO_2_ content in the shell. The bicomponent multifilament yarns with 3 wt% TiO_2_ (thin shell) were most effective in the bacterial inhibition due to the greater deposition of TiO_2_ nanoparticles on and near the shell surface.

Natural compounds from plants, vegetables, fruits, or other natural resources are rich in various bioactive compounds such as flavonoids, and alkaloids [16]. These compounds demonstrate various biological effects such as antibacterial, anti-inflammatory, or antioxidant effects. Their effect (i.e., biological activity) can be used in the medical field by incorporating these materials in medical textiles [17]. Natural compounds are already added to polymer film such as coffee, cacao, basil oil extract, oregano, and cinnamon oil [18,19,20,21]. However, not enough research has been conducted on the compounds that could be used in the melt-spinning process.

Sharifah et al. [22] investigated an opportunity to form PLA fibers with curcumin. It was noted that 5% curcumin-loaded fibers have lower mechanical properties (tensile strength and modulus) compared to pure PLA fibers. One of the reasons for this is the lack of internal bonding in the PLA fiber. Gaidukovs et al. [23] investigated the influence of amber fillers on the mechanical and chemical properties of polyamide 6 (PA6) multifilament yarns. They determined that the PA6 multifilament with amber particles have inferior mechanical properties. For example, a PA6/amber blend with a tenacity of 16.35 cN/dtex was less than the pure PA6 filament tenacity of 32.09 cN/dtex. Nevertheless, the formatted yarns are strong enough to be used in medical textiles. Belkhir and co-authors [24] investigated using melt-spinning methods by incorporating 5% hydrolyzed casein into the polymer matrix of polypropylene (PP). Weaker mechanical properties (tenacity) of multifilaments with hydrolyzed casein were observed in comparison with pure PP multifilaments. This can be attributed to the poor adhesion of the PP matrix with hydrolyzed casein causing structural defects. Multifilaments loaded with 5% hydrolyzed casein showed high efficiency as an antibacterial material. After the antibacterial test, it was found that the antibacterial activity of 5% hydrolyzed casein in PP multifilament value was higher than 6.4, compared to other studies that showed that in the presence of silver and ZnO, antibacterial activity had a value of 4.5 against *S. aureus*.

Colophony, also known as rosin, is a solid fraction of the fresh resin obtained from pines after heating to vaporize the volatile liquid terpene components. It is insoluble in water and soluble in most organic solvents. Rosin is composed of 10–20% neutral compounds (diterpenoid alcohols, aldehydes, and hydrocarbons) and 80–90% of resin acids (mostly containing abietane- or pimarane-type structures). Rosin derivatives demonstrate an antibacterial activity against *E. coli*, *S. aureus*, and *B. subitilis* and an antifungal effect on *V. mali*. Moustafa and co-authors estimated that rosin and PLA/poly(butylene adipate-co-terephthalate) (PBAT) polymer film showed an antibacterial effect [25,26,27,28,29]. Due to the fact of these properties, rosin composites can be used for biomedical applications. Rosa-Ramírez and co-authors [30], modified PLA by melting the compound with gum rosin (GR) and investigated the mechanical and thermal performance of a polymer film. Kanerva et al. [31] formed multifilaments from PLA and polyethylene polymers with two different rosin concentrations (0% and 10%) using a Masterbatch method. It was estimated that after the formation of PLA multifilaments with 10% rosin, they had lower mechanical properties (i.e., tensile strength and elongation at break) than pure PLA multifilaments. Tensile strength decreased by approximately 66% and elongation at break decreased by approximately 88%. Natural compounds such as those listed above can provide suitable properties for multifilament yarns. This would be useful in the development of biomedical textiles and would be a good alternative to metal nanoparticles and their oxides.

The aim of this research was to investigate the formation of biodegradable melt-spun multifilament PLA yarns with added rosin and to determine the influence of different contents of rosin on the structure, mechanical, thermal, optical, and surface wetting properties of these yarns.

## 2. Materials and Methods

### 2.1. Materials

The polylactic acid 6100D (Nature Works, Blair, NE, USA) with an average molecular weight (M_w_) of nearly 190 kDa was used as the main material for the formation of multifilament yarns. Its density was 1.24 g/cm^3^, glass transition temperature reached 55–60 °C, and its melting temperature varied from 165 to 180 °C [32]. Pine rosin was imported from “Mungolux” (Germany). It was 100% pure rosin without any additives and with a melting point T_m_ = 100 °C. Ethanol (96%) was used as the solvent for pine rosin extract formation.

### 2.2. Preparation of Pine Rosin Solution in Ethanol

Solid rosin particles were crushed to fine powder for better solubility. The mass ratio of rosin and ethanol in solution was 1:1 wt/wt. The solution was stirred with a magnetic stirrer at 400 rpm (IKA RH, basic KT/C, Staufen, Germany) until all the rosin was dissolved.

### 2.3. Modification of PLA Granules with Pine Rosin

The PLA granules were modified with a rosin ethanolic solution through a spraying process. PLA granules were sprayed with an ethanolic rosin solution (50% wt/wt), mixed with a glass rod in a Teflon dish and dried at a temperature of 80 °C for at least 60 min until the ethanol evaporated. This procedure was repeated several times until PLA granules with different amounts of rosin (i.e., 5%, 10%, and 15%) were obtained.

### 2.4. Melt-Spinning of PLA Multifilament Yarns

**Figure 1:**
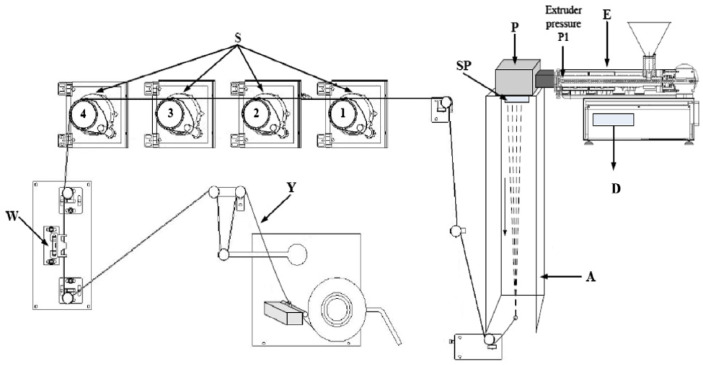
Principal scheme of the laboratory spinning equipment COLLIN ^®^ CMF 100 (Collin GmbH, Germany): E—extruder, P—melting pump, SP—spinneret, A—air quench cabinet, D—display, S—stretching gadgets, W—whirling unit, and Y—multifilament yarn from microfibers [33].

Multifilament yarns from PLA pure polymer and PLA modified with rosin ethanolic solution were manufactured using a COLLIN^®^ CMF 100 (Collin GmbH, Maitenbeth, Germany) single-screw extruder. The single-screw extruder (L/D ratio of 25:1) had seven heating zones, where the temperature during the experiments was set to 180 °C. The average speed of the extruder was set to 29 rpm. The circular spinnerets (Figure 1, indicated by SP) with 24 holes (0.45 mm diameter) were used during these experiments. Cooling of the filaments (Figure 1 indicated by A) was achieved through cross-flow air quenching at a temperature of 14 °C. The temperatures of the stretching rolls were as follows: S1–S4 = 55 °C in all experiments. Multifilament yarns from the polymers were formed by changing the melt-spinning parameters, which are given below in Table 1.

### 2.5. Linear Density of Yarns

The linear density of yarns was measured based on the previous work method [34]. For the tests, PLA and PLA/rosin multifilament yarns were conditioned for no less than 24 h at the standard atmosphere according to the standard, i.e., at a relative humidity of (φ = 65% ± 4%) and a temperature of 20 ± 2 °C. The specimens of 50 m in length were prepared by reeling skeins with a Zweigle L232 (Zweigle Textilprüfmaschinen GmbH & Co. KG, Reutlingen, Germany) in order to estimate the linear density of multifilament yarns. The mass of the skeins was determined under the standard atmospheric conditions with laboratory scales KERN EW150-3M (Kern & Sohn GmbH, Balingen, Germany). The linear density of multifilament yarns was calculated according to the equation:

(1) T=m/l, tex or g/km.

where m—mass, and g and l—length of specimen, km.

The test result was calculated based on the average of five measurements.

### 2.6. Structure of PLA Multifilament Yarns

The structure of PLA multifilament yarns was determined using a scanning electron microscope SEM S-3400N (Hitachi, Tokyo, Japan (beam voltage: 3 kV, magnification: 50×, scale bar: 1 mm)). The diameter of the fiber was evaluated using SEM images and the software NIS-Elements D (Nikon Corporation, Tokyo, Japan). The average of 140 microfiber diameters was calculated using the measurements of the SEM images.

### 2.7. Mechanical Properties of PLA Multifilament Yarns

Mechanical properties (tenacity (cN/tex), breaking tenacity (cN/tex), tensile strain (%), and elongation at break (%)) of PLA multifilament yarns were determined according to the EN ISO 2062:2009 standard. These experiments were carried out under a standard atmosphere, where the temperature was 20 ± 2 °C and the relative humidity was 65 ± 4%. A universal testing equipment, Zwick/Roell (Zwick GmbH & Co. KG, Ulm, Germany), with the operating program testXpert^®^ were used. The length between the clamps was 250 mm, and the stretching speed was 500 mm/min with a pretension of 0.5 cN/tex. The number of tensile tests for the package was 35.

### 2.8. Optical Properties of PLA Multifilament Yarns

The UV–Vis diffuse reflectance spectra of melt-spun multifilament yarns were measured with a Lambda 35 UV/VIS spectrometer (Perkin–Elmer, Waltham, MA, USA) at a wavelength ranging from 200 to 800 nm. Diffuse reflectance measurements of PLA multifilament yarns were performed by wrapping multiple layers (no less than 10) around a glass coverslip typically used in microscopy.

### 2.9. Thermal Behavior of PLA Multifilament Yarns

Differential scanning calorimetry (DSC) analysis was carried out using a Netzsch Polyma DSC 214 (NETZSCH-Gerätebau GmbH, Selb, Germany). It was conducted on pure PLA and PLA/rosin multifilament yarns to obtain the glass transition, melting, crystallization, and cold crystallization temperatures of the specimens. Aluminum pans were used for each sample with an average sample mass of 5 mg. The scan rates in both heating and cooling modes were 10 °C/min, under a nitrogen atmosphere at a flow rate of 20 mL/min. For each example, the procedure was as follows: the first heating scan at 10 °C/min from 15 up to 240 °C, an isotherm at this temperature for 3 min, then a scan at 10 °C/min down to 15 °C, an isotherm at this temperature for 3 min, and, finally, the second heating scan from 15 to 240 °C at 10 °C/min. The first cycle was the most important, because it was influenced by the thermal history of the material and changed over time with degradation. The degree of crystallinity of pure PLA and PLA saturated with rosin samples was calculated using the following equation [35]:

(2) $$x_{c}\left(\%\right)=\frac{\Delta H_{m}-\;\Delta H_{c}}{\lambda \Delta H_{m,o}}*100\%$$

where ΔH_m_—melting enthalpy of PLA (J/g); ΔH_c_—cold crystallization enthalpy of PLA (J/g); λ—mass fraction of PLA in composite yarns; ΔH_m,o_—melting enthalpy of 100% crystal; the value was 93.6 J/g [36].

### 2.10. Chemical Interactions between PLA and Rosin

The Raman scattering measurement was performed using an inVia Raman microscope (Renishaw, Kingswood, UK). In order to record the spectra, an exciting wavelength (λ = 633 nm) was used which was provided by a helium–neon laser. All peaks were obtained using a 50× lens. The excitation beam from a diode laser with a wavelength of 532 nm was focused on the sample using a 50× objective (NA = 0.75, Leica). The laser power on the sample surface was 0.15–0.3 mW. For all measurements, the integration time was 10 s. The Raman Stokes signal was dispersed using a diffraction grating (2400 grooves/mm), and data were recorded using a Peltier-cooled charge-coupled device (CCD) detector (1024 × 256 pixels). This system yields a spectral resolution of approximately 1 cm^−1^. Silicon was used to calibrate the Raman setup in both Raman wavenumber and spectral intensity.

### 2.11. Liquid Contact Angle (CA) of PLA/Rosin Multifilament Surface Analysis

**Figure 2:**
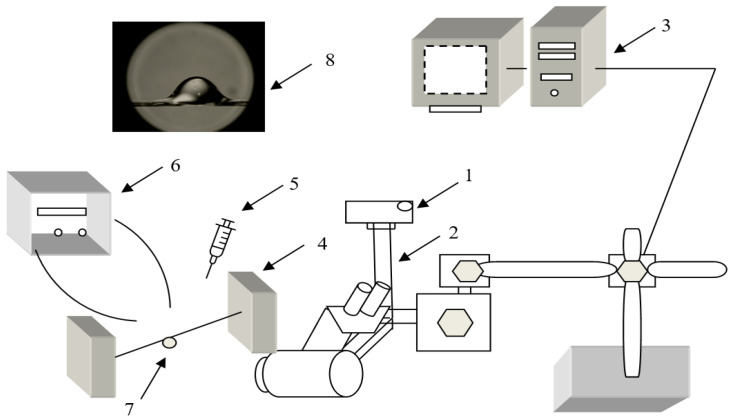
Principal scheme of absorbency test: (1) stereoscopic microscope; (2) digital camera; (3) computer; (4) yarn anchorage system; (5) pipette; (6) light source; (7) drop of liquid on the yarn; (8) picture of the video record.

The wettability of yarns can be characterized by the contact angle. The static testing drop method was used in this study. Absorbency tests using the drop method were carried out using two types of liquids: physiological saline (B. Braun Melsungen AG, Hessen, Germany) and glycerol (Sigma–Aldrich, Chemie GmbH, Taufkirchen, Germany). This test method is designed to measure the liquid absorbency of yarns by measuring the time it takes for a drop of liquid placed on the yarn surface to be completely absorbed into the yarn. (Figure 2). A droplet of ~9 µL was dripped onto the yarn’s surface from a height of 0.2 cm to the needle. A stopwatch was started as soon as the drop fell on the yarn and was stopped as soon as the image of the reflected light disappears at the edge of the drop, i.e., the liquid drop was completely absorbed by the yarn or the variation is irrelevant. This is termed as the drop absorbency time. The process was recorded by a stereo microscope (Nikon Stereoscopic Microscope SMZ 800) and a digital camera Nikon Coolpix 4500 connected to a computer. The changes in fluid angles and the time were measured using the NIS-element and were compared with the initial wetting angles at the initial observation time of 0 s. The average liquid absorption time was calculated using the data of five individual measurements.

### 2.12. Statistical Analysis

To investigate the influence of rosin added on the linear density of melt-spun PLA multifilament yarns and their mechanical properties, the differences between average values of various parameters were estimated by the Student’s *t*-test. The level of significance was set at *p* < 0.05. For comparing the mechanical properties of two experimental results, the Student’s *t*-test was also used; if *t*_ɑ_ obtains *t*_ɑ_ < *t*_95_, then result is insignificant; however, if *t*_ɑ_ < *t*_99_, then the result is significant. *t*_95_ = 1.99 and *t*_99_ = 2.65.

## 3. Results and Discussion

### 3.1. The Influence of Rosin on the Linear Density and Mechanical Properties

**Figure 3:**
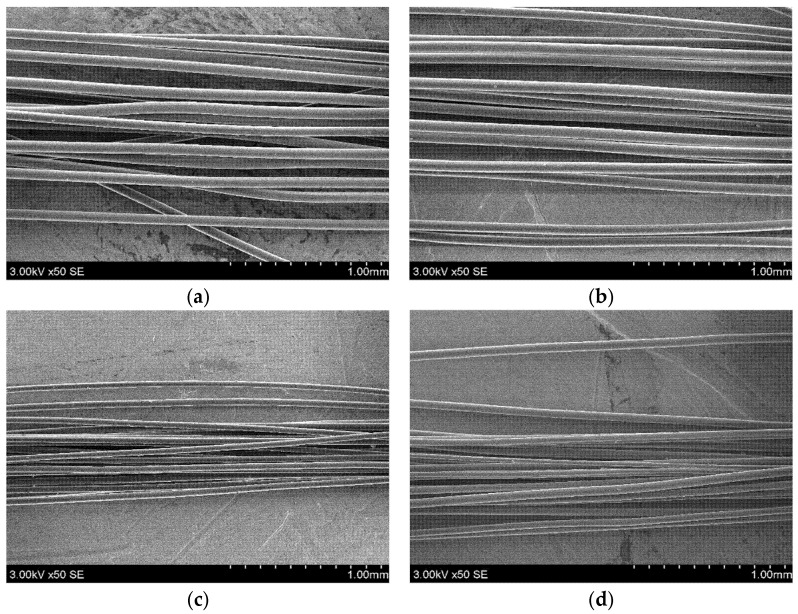
SEM images of (**a**) PLA with 5% rosin and a 1.75 draw ratio; (**b**) PLA with 10% rosin and a 1.75 draw ratio; (**c**) PLA with 5% rosin and a 2.75 draw ratio; (**d**) PLA with 10% rosin and a 2.75 draw ratio.

In this study, PLA multifilament yarns were formed with two different rosin concentrations (i.e., 5% and 10%) and two different draw ratios (i.e., 1.75 and 2.75). Using PLA granules modified with 15% rosin, multifilament yarns did not form due to the separation of the melt phases during the formation. The PLA multifilament yarns formed with 5% and 10% rosin concentrations and 1.75 and 2.75 draw ratios had smooth and solid structures (Figure 3).

**Figure 4:**
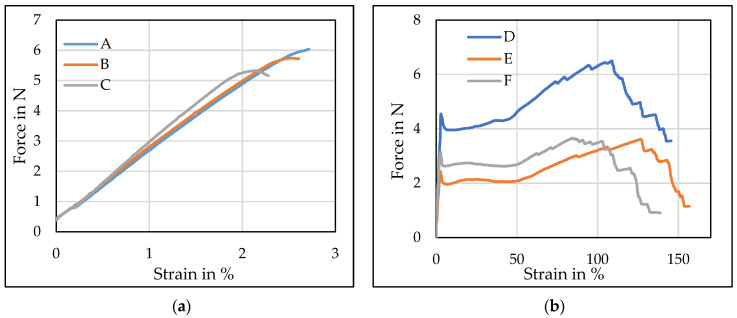
Typical stress–strain curves of the formed multifilament yarns with (**a**) a 1.75 draw ratio; (**b**) a 2.75 draw ratio.

Linear density and the tensile tests on the formed PLA multifilament yarns were analyzed in order to determine how the mechanical properties were influenced by the presence of rosin in PLA. On yarns formed at two different draw ratios, 1.75 and 2.75, the data on the linear density (tex), mechanical properties: tenacity (cN/tex), breaking tenacity (cN/tex), and tensile strain (%) and elongation at break (%) of pure PLA multifilaments and PLA with rosin are presented in Table 2, and the typical stress–strain curves are presented in Figure 4.

The linear density (tex) of melt-spun PLA yarns depends on the different technological parameters (melt temperature, draw, and pressure) [13,34]. If the draw ratios increase, the linear density of the PLA multifilament yarns decrease (34–39%), all other spinning variables remaining constant. Similar results were observed by Gajjar, Twarowska-Schmidt, and Zakir Hossain [34,37,38]. The draw ratio had a significant influence on the properties of the melt-spun yarn due to the changes in the microstructure (polymer alignments and crystallinity) as well as the diameter [37]. Due to the poorer alignment of pure macromolecules, the PLA multifilament yarns A, B, and C, formed at a lower draw ratio (1.75), exhibited high fragility.

From the data presented in Table 2, it noticeable that the presence of rosin had a significant effect on the linear density of melt-spun PLA yarns, when yarns are formed at a lower (1.75) draw ratio (i.e., A–C). A higher draw ratio (i.e., D–F) had no significant influence on the linear density of PLA multifilament yarns. Comparing the data on the average linear density according to the Student’s *t*-test, it was estimated that the difference between the average linear density was significant, comparing A–C. Comparing D–F, the difference was insignificant. During the draw process of the PLA multifilament yarns with the drawing rollers at 55 °C, it is easier to draw PLA filaments with the added rosin, then when the draw ratio was only 1.75. When the draw ratio was 1.75, the PLA multifilament yarns with higher linear density (Table 2) and a diameter of single filaments (Table 3) were formed. The alignment of macromolecules of such filaments was low, and the rosin worked as a plasticizer that facilitated the PLA chain’s mobility, which improves filament drawability. Such results correlate with the works of other authors [39,40,41,42,43]: that a low plasticizer concentration decreases the linear density of multifilament yarns when used under the same conditions of formation and, consequently, multifilament yarns with lower linear density and diameter are formed (comparing A with B and C). When the draw ration of PLA multifilament yarns is 2.75, the alignment of the macromolecules of filaments is higher and multifilament yarns with a lower linear density and thinner filaments are formed (see Table 2). It is possible to make an assumption that due to the small amount of rosin in the E and F yarns, the drawability of multifilament yarns did not improve and the linear density did not change significantly.

Tensile tests on PLA melt-spun yarns were estimated to determine how the mechanical properties of yarns were influenced by different draw ratios and the presence of rosin in PLA. In Figure 3, it is noticeable that the tensile curves of the multifilament yarns formed at a draw ratio of 1.75 (Figure 3a) and 2.75 (Figure 3b) are completely different. It was already mentioned before that when the draw ratio was 1.75, the PLA multifilament yarns (i.e., A–C) increased their fragility (due to the arrangement of small macromolecules), which was not observed in PLA multifilament yarns at a 2.75 draw ratio (i.e., D–F). Due to the fragility of PLA multifilament yarns, the tenacity (cN/tex) and tensile elongation (%) of the A, B, and C multifilament yarns were very similar to the breaking tenacity (cN/tex) and breaking elongation (%) (Table 2). Multifilament yarns, which have a higher molecular arrangement (D–F), gradually broke during the tensile tests. Consequently, the breaking tenacity (cN/tex) was ~74% lower than tenacity (cN/tex), and tensile elongation (%) was ~30% lower than breaking elongation (%). Similar results were submitted by F. Mai with co-authors [44]. They estimated that at a low draw ratio Poly(l-lactic acid) tapes excel fragility, but when the draw ratio is 2 and higher, elongation increases due to the better molecular orientation.

PLA multifilament yarns, formed at a draw ratio of 1.75 (i.e., A–C), had approximately ~48% lower tenacity (cN/tex) due the nonaligned nature of the molecules compared to the multifilament yarns that were formed at a draw ratio of 2.75 (i.e., D–F). The presence of 10% rosin in PLA reduced the tenacity (cN/tex) of the PLA multifilament yarns by 7% and 27% as well as the breaking tenacity by 7.5% and 27% at a draw ratio of 1.75 and 2.75, respectively (Table 2). Based on the Student’s *t*-test, different draw ratios as well as the presence of rosin in PLA had a significant influence on the tenacity (cN/tex) and breaking tenacity (cN/tex) of multifilament yarns.

A 10% presence of rosin in PLA decreased the tensile strain by 12% and the breaking elongation of PLA multifilament yarns by 8% at a draw ratio of 1.75 and increased the tensile strain by 5% and breaking elongation by 4% at a 2.75 draw ratio. The decrease in mechanical properties, such as tenacity and elongation at break when used at 1.75 draw ratio, was caused by the lack of a molecular orientation leading to the observed nonaligned nature of the molecules. The same results were estimated by Cicero [45]. According to De La Rosa-Ramírez and Li [30,46], low contents of rosin excel the plasticizing effect of the PLA polymer matrix. Increasing the rosin concentration, the breaking force is associated with the beginning of a separation phase due to the saturation effect, which weakens the polymer by generating stress concentration. Thus, it can be assumed that PLAs modified with 5% rosin have a plasticized polymer chain structure and increased elongation at break. However, a higher concentration of rosin decreases elongation at break and lowers significant results compared with pure PLA multifilament yarns.

### 3.2. Optical Analysis of Melt-Spun Multifilament Yarns

**Figure 5:**
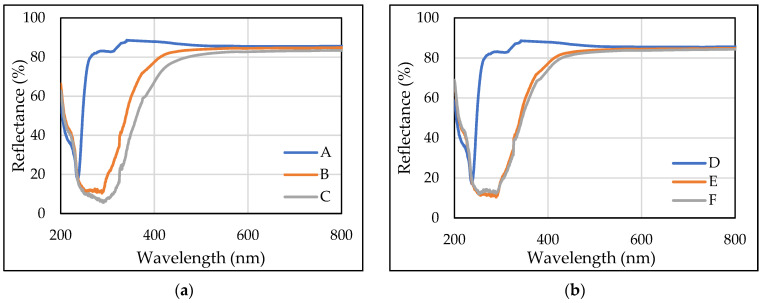
UV–Vis spectra analysis of PLA multifilament yarns with (**a**) a 1.75 draw ratio; (**b**) a 2.75 draw ratio.

The light reflectance curves obtained by UV–Vis spectroscopy are presented in Figure 5, which shows that the draw ratio and concentration make no difference because melt-spun multifilament yarns with rosin demonstrate the lowest reflectance (and the highest absorbance) compared with pure PLA multifilament yarns. Figure 5 also shows that pure PLA multifilament yarns have the lowest absorbance in the whole visible and near UV regional spectrum. The reflectance decreased from 340 nm for the entire UV region spectra. The lowest reflectance peak was at a 240 nm wavelength. This peak was observed in all spectra (Figure 5a,b). It was caused by the strong absorption of UV radiation by the ester groups of polylactide chains [47].

The presence of compounds introduced into PLA multifilament yarns through the addition of rosin (i.e., B, C, E, and F) indicates a significant increase in absorption (and a decrease in diffuse reflection) in the wavelength ranging from 500 to 400 nm as well as broad and intensive absorption bands in the entire UV region (from 200 to 400 nm) of the recorded spectra. Comparing different draw ratios in multifilament with rosin, it can be seen that all trends of the curves are similar. At a 1.75 draw ratio and different rosin concentrations, the PLA multifilament yarns with 10 wt% had lower reflectance compared with PLA multifilament yarns with 5 wt% in the spectral range from 250 to 550 nm. Comparing the results when using a 2.75 draw ratio, it is evident that there was no difference between different concentrations of rosin. Comparing the spectra and yarn appearances, differences between pure PLA multifilament yarns (i.e., A and D) and PLA with rosin additive multifilament yarns (i.e., B, C, E, and F) were observed, which were most likely determined by the rosin. The modified PLA multifilament yarns (i.e., B, C, E, and F) were characterized by decreased reflection, a consequence of rosin having light absorbing agents [47].

It should be noted that the significant decrease in diffused reflectance (and the highest absorbance) was characteristic of multifilament yarns made from PLA modified with 10% rosin concentration (i.e., C and F), and this must be related to a higher concentration of rosin present. Similar results can be seen in multifilaments containing natural compounds with myrrh and polymeric film containing cinnamon oil, myrrh, and rosin. After modifying multifilament yarns with natural compounds, the films also showed lower reflectance in the UV region [17,36,47].

### 3.3. Thermal Behavior of PLA Melt-Spun Multifilament Yarns

**Figure 6:**
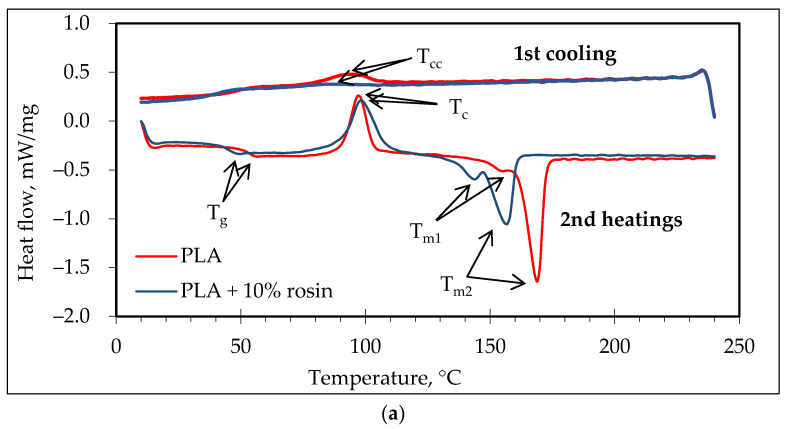

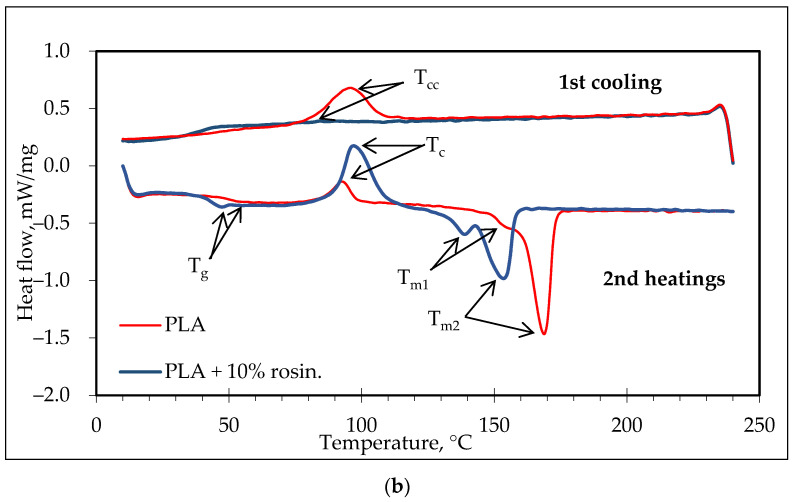
DSC thermogram analysis of (**a**) multifilaments when the draw ratio was 1.75; (**b**) multifilaments when the draw ratio was 2.75.

Figure 6 shows the DSC curves for PLA and PLA with 10% rosin in multifilament yarns. The results obtained from the DSC curves for both pure PLA and PLA with rosin are summarized in Table 4, which shows that the different draw ratios of multifilaments (A and D) and (C and F) demonstrated a similar thermal behavior. The PLA/rosin multifilament yarn sample demonstrated the differences between the glass transition temperature of pure PLA multifilament T_g_ (51.2–50.7 °C), and then PLA containing rosin T_g_ decreased to (47.3–47.9 °C). The highest difference was observed when the melting point temperatures were compared. When PLA was modified with rosin, the melting points T_M1,2_ dramatically decreased—T_M1_ from 155.4 to 138.8–143.7 °C and T_M2_ from 168.9 C to 153.4–156.5 °C. By analyzing the thermograms of melt-spun multifilament yarns, it is possible to observe that multifilament yarns from PLA modified with rosin (i.e., C and F) had the lowest degree of crystallinity, X_c_. Glass T_g_ and melting temperatures (T_m_) were lower for PLA/rosin yarns. The lower T_g_ was attributed to either lubrication or a plasticizing effect caused by the rosin added to the PLA polymer matrix, which explains the higher mobility of polymer chains at lower temperatures [48]. The exothermal cold crystallization temperature (T_c_) for pure PLA was lower when compared to PLA/rosin multifilament (i.e., D and F). One of the reasons for a higher T_c_ of PLA/rosin is that the addition of rosin slows down the migration of PLA chains towards the nucleus surface. The slower migration of PLA chains is, in turn, associated to the enhanced interaction between rosin and PLA so that the diffusion rate of PLA chains is affected by the presence of rosin [47].

DSC cooling curves (Figure 6a,b) show that rosin had a significant influence on the peak crystallization temperature when cooling (T_cc_) multifilament yarns. Pure PLA multifilament yarns had a clear peak T_cc_ compared with PLA modified with rosin. The reason why the peak of cold crystallization disappears is that rosin affects the integrity of the PLA crystal and prevents the regular accumulation of molecular chains. This is corroborated and proved by the lower crystallinity (X_c_) of PLA/rosin compared to pure PLA. These results correlate with the works of other authors [47,48], where it was estimated that high concentrations of additives reduce PLA’s ability to form the normal polycrystalline structure of the polymer, As a result, there is a decrease in the crystallinity of the composite material.

### 3.4. Raman Spectroscopy

**Figure 7:**
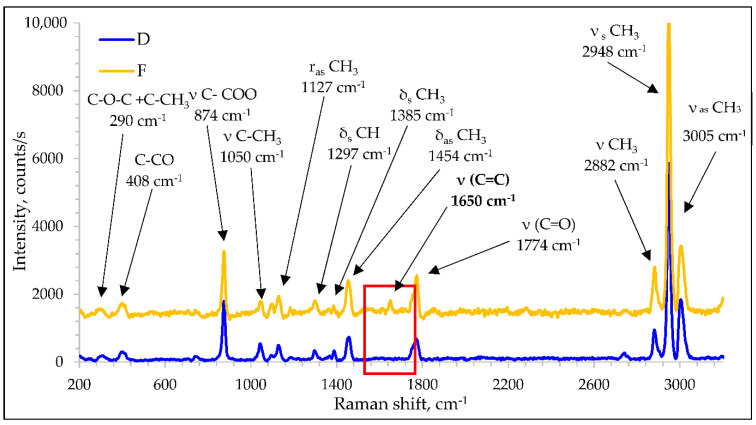
Raman spectra of PLA and PLA/rosin.

The Raman spectra of pure PLA multifilament yarns and PLA modified with rosin for the main bands and observed wavenumbers are shown in Figure 7. Comparing pure PLA and PLA modified with rosin, typical peaks of PLA were identified. Peaks at 3005, 2948, and 2882 cm^−1^ were assigned to the. CH_3_ stretching vibration: asymmetric stretching 2995–3002 cm^−1^, symmetric stretching 2944–2947 cm^−1^, and 2878–2889 cm^−1^ modes [48]. The second peak of pure PLA was C=O stretching at 1774, 1454, and 1385 cm^−1^, and there were symmetric and asymmetric deformation modes of the CH_3_ groups. The peak at 1297 cm^−1^ could be due to the CH deformation vibration peak that is usually found at 1299–1305 cm^−1^. The peak at 1127 cm^−1^ can be assigned to the CH_3_ group rocking vibration, the peak at 1050 cm^−1^ to the stretching of C–CH_3_ bond, the peaks at 874 cm^−1^ and 408 cm^−1^ to the vibrational states of the C–COO and C–CO groups, respectively. The peak at 308 cm^−1^ was characteristic of the vibration of two different groups: C–O–C and C–CH_3_ [49].

The spectrum of the PLA multifilaments with 10% rosin contained all polymer-specific bands that were observed in the pure PLA multifilament yarn spectrum as well as the presence of an additional band around 1650 cm^−1^ which corresponded to the ν(C = C) stretching vibrations. This band can be attributed to the structure of rosin-based acids, of which abietic acid (C_19_H_20_COOH) is predominant. The Raman spectra of rosin-based acids contain features that can be attributed to typical bands in the range of 1600–1800 cm^−1^ [50]. It can be clearly stated that rosin acids are present in the formed PLA multifilament yarns.

### 3.5. Contact Angle (CA) of PLA/Rosin Multifilament Analysis

Table 5 shows the results of different liquid contact angles. Samples with higher linear density (lower draw ratio: A–C) had a lower physiological saline contact angle compared with the D, E, and F samples, where the draw ratio was higher, 2.75. PLA multifilament yarns A, B, and C had an approximately three times higher linear density, so when multifilament yarns have a higher linear density, a drop has a bigger contact area, and it can better wet the multifilament yarn surface.

The contact angles of the physiological saline droplet on the A and D samples were the smallest (i.e., 51.09° and 62.72°) compared with multifilament yarns containing rosin. The samples with the highest rosin concentrations (i.e., C and F) had higher (i.e., 19% and 7%) contact angles of physiological saline drops.

The contact angles of glycerol droplet of the remaining samples were relatively larger compared with the contact angle results of the physiological saline. One of the reasons for this is that glycerol has a slightly higher surface tension (i.e., 76.2 mN/m) than physiological saline (i.e., 73.9 mN/m). The second reason why the absorption time of a glycerol drop on the D–F yarns were the longest compared with the A–C yarns was the different yarn morphologies (i.e., higher linear density).

Thus, the highest glycerol and physiological saline contact angles were noted on the multifilaments with the highest rosin content; thus, the presence of rosin in multifilament yarns reduces their wettability. Similar results were estimated by Yang and co-authors, when they analyzed rosin’s influence on wood surface wettability and indicated that a rosin coating had a strong influence on reduced surface wettability [49].

## 4. Conclusions

Multifilament yarns containing pure PLA and PLA with different concentrations of rosin were melt-spun. The results of this study confirmed that it is possible to melt-spun PLA multifilament yarns with rosin. In the Raman spectra, the presence of the rosin acidic compound in the polymer matrix was indicated. The draw ratio of 1.75 was too small for the formation of strong PLA multifilament yarns due to the assumed poor orientation of small macromolecules and such yarns exhibit brittleness.

The presence of rosin in PLA impacted on the mechanical, optical, and thermal properties as well as wetting behavior of melt-spun yarns. Melt-spun yarns with rosin had poorer (or inferior) mechanical properties (lower tenacity (cN/tex) and breaking tenacity (cN/tex)) and lower melting temperature and degree of crystallinity when compared to pure PLA multifilament yarns. Rosin increased the tensile strain (%) and elongation at the break (%) and reduced PLA’s ability to form the normal paracrystalline structure of the polymer. Modified PLA polymer with rosin produced multifilament yarns that demonstrated the highest absorbance in the whole UV region compared with the pure PLA multifilament yarns. The analysis of the contact angles of the multifilament yarns showed that multifilament with 10% rosin was more hydrophobic than pure PLA multifilament yarns.

## Figures and Tables

**Table 1:** Parameters for spinning to obtain multifilament yarns.

Code	Samples	Stretching Rolls Speed Rpm				Draw Ratio
		S1	S2	S3	S4	
A	PLA	80	100	120	140	1.75
B	PLA + 5% Rosin					
C	PLA + 10% Rosin					
D	PLA	130	210	288	358	2.75
E	PLA + 5% Rosin					
F	PLA + 10% Rosin					

**Table 2:** Mechanical properties of the formed multifilament yarns.

Code of Sample	Linear Density of Multifilament Yarns ± Δ * (tex)	Tenacity ± Δ * (cN/tex)	Breaking Tenacity ± Δ * (cN/tex)	Tensile Strain ± Δ * (%)	Elongation at Break ± Δ * (%)
A	151.2 ± 2.0	4.2± 0.1	4.0 ± 0.1	2.5 ± 0.1	2.6 ± 0.1
B	128.9 ± 1.8	4.3 ± 0.1	4.2 ± 0.1	2.5 ± 0.1	2.7 ± 0.1
C	131.7 ± 2.2	3.9 ± 0.1	3.7 ± 0.1	2.2 ± 0.1	2.4 ± 0.1
D	51.7 ± 2.4	10.0 ± 0.2	2.6 ± 0.1	98.4 ± 3.8	145.1 ±3.2
E	50.7± 1.1	7.2 ± 0.2	1.7 ± 0.2	126.0 ± 11.6	172.5 ± 6.5
F	49.4 ± 2.5	7.3 ± 0.1	1.9 ± 0.1	103.7 ± 2.4	150.8 ± 3.8

* Δ—random error.

**Table 3:** Diameter of melt-spun multifilament yarns.

Sample Code	A	B	C	D	E	F
Diameter, µm	80.1 ± 2.7	74.5 ± 1.5	76.2 ± 1.1	47.7 ± 1.6	47.5 ± 0.9	47.8 ± 1.5

**Table 4:** DSC analysis of pure PLA and modified PLA with rosin multifilament yarns *.

Code of Sample	T_g_ (°C)	T_c_ (°C)	∆H_cc_ (J/g)	T_m1_ (°C)	T_m2_ (°C)	T_cc_ (°C)	∆H_m_ (J/g)	Crystallinity X_c_ (wt%)
A	51.20	97.20	27.03	155.40	168.90	93.20	55.85	30.60
C	47.30	96.80	47.43	138.80	153.40	84.50	48.70	1.30
D	50.70	92.50	10.59	155.40	168.80	95.70	57.11	49.50
F	47.90	98.30	42.20	143.70	156.50	84.10	47.27	5.30

* First heating scan at 10 °C/min from 15 up to 240 °C, the second heating scan from 15 to 240 °C at 10 °C/min.

**Table 5:** The values of contact angle of liquid on yarns.

Contact Angle Value *θ*, deg ± Δ										
Liquid	Physiological Saline	Glycerol	Physiological Saline	Glycerol	Physiological Saline	Glycerol	Physiological Saline	Glycerol	Physiological Saline	Glycerol
Time	0		10		20		60		120	
Samples										
A	51.1 ± 0.3	62.2 ± 4.5	35.2 ± 2.0	54.3 ± 5.4	-	47.4 ± 3.2	-	44.3 ± 4.0	-	43.1 ± 2.1
B	52.9 ± 0.8	64.3 ± 3.7	36.7 ± 3.1	56.4 ± 1.8	-	51.7 ± 2.2	-	47.3 ± 3.7	-	43.6 ± 2.7
C	60.7 ± 2.8	79.3 ± 6.1	50.8 ± 2.3	69.9 ± 5.1	29.8 ± 3.3	67.9 ± 5.8	-	66.4 ± 5.1	-	64.5 ± 4.9
D	62.7 ± 4.6	84.3 ± 2.2	55.7 ± 5.1	83.4 ± 2.3	51.6 ± 4.8	83.0 ± 2.9	32.1 ± 2.8	82.5 ± 3.2	-	80.7 ± 2.8
E	65.4 ± 5.6	86.2 ± 0.9	57.2 ± 2.0	81.6 ± 5.5	51.8 ± 5.1	81.6 ± 5.9	36.2 ± 5.4	79.5 ± 7.0	-	78.3 ± 3.9
F	67.1 ± 3.7	88.7 ± 3.7	57.2 ± 2.1	88.0 ± 4.2	51.2 ± 4.6	87.9 ± 4.4	34.8 ± 2.3	86.7 ± 5.4	-	86.1 ± 3.1
